# A global inventory of methane emissions from abandoned oil and gas wells and possible mitigation pathways

**DOI:** 10.1093/nsr/nwaf184

**Published:** 2025-05-19

**Authors:** Tianyang Lei, Xiujing Chen, Shijun Ma, Liang Jing, Dabo Guan

**Affiliations:** Department of Earth System Sciences, Tsinghua University, Beijing 100080, China; The Bartlett School of Sustainable Construction, University College London, London WC1E 6BT, UK; Department of Earth System Sciences, Tsinghua University, Beijing 100080, China; The Bartlett School of Sustainable Construction, University College London, London WC1E 6BT, UK; Energy Sustainability Analysis, Technology Strategy and Planning Department, Saudi Aramco, Dhahran 31311, Saudi Arabia; Department of Earth System Sciences, Tsinghua University, Beijing 100080, China; The Bartlett School of Sustainable Construction, University College London, London WC1E 6BT, UK

**Keywords:** abandoned oil and gas wells, methane emission, methane-reduction pathway

## Abstract

Methane emissions from abandoned oil and gas (AOG) wells are significant but poorly quantified, contributing to uncertainties in global greenhouse gas inventories and hindering progress toward net-zero emission targets. In fact, the actual level of methane emissions from AOG wells worldwide and the geological characteristics of the wells remain poorly understood. Here, we develop a resource-type-specific, comprehensive inventory of methane emissions from 4.5 million AOG wells across 127 countries as of 2022, including detailed well-level data for 420 000 wells (9% of the total). We estimate global methane emissions from AOG wells at 0.4 Mt in 2022. Historical cumulative emissions are estimated at 11.2–12.6 Mt, of which 90% originate from unplugged wells. Over the period 2023–50, methane emissions from the AOG wells included in our inventory are projected to be 9.9 Mt in the absence of any decarbonization intervention (slightly higher than Spain's annual greenhouse gas emissions in 2019). Plugging abandoned wells is critical to reducing emissions, with potential mitigation of 53%–61% over 2023–50. High-emitting wells—particularly those tied to major oil and gas producers—should be prioritized. This study provides refined estimates of methane emissions and possible mitigation pathways for global AOG wells at well/country levels that have been overlooked in global greenhouse gas emissions inventories.

## Teaser

This study presents the first global inventory of methane emissions from 4.5 million abandoned oil and gas wells, revealing major overlooked emission sources and tailored mitigation opportunities.

## INTRODUCTION

With a warming impact that is 84 times greater than CO₂ over a 20-year period and 28 times greater over 100 years [1], methane is the second-most powerful greenhouse gas behind CO_2_ [2,3] and represents a crucial target in the context of emissions reductions over the next two decades. According to the intergovernmental panel on climate change (IPCC) report on global warming of 1.5°C [4], reducing methane immediately along with other climate-forcing gases is necessary to achieve the Paris Agreement.

Abandoned oil and gas (AOG) wells, i.e. inactive wells with no production (see details of the definition in ‘Materials and method’), are potential sources of methane emissions, even though with large uncertainty [5,6]. An abandoned well [7], even when plugged appropriately, has the potential to permanently leak methane [8], not to mention that millions of inactive wells worldwide are abandoned without proper measures (plugging measures, regular monitoring, surface restoration, etc.). These conditions present both a significant emission risk and an opportunity for surface-based mitigation.

Methane emissions from AOG wells have been assessed previously at the state level [5–7,9,10], regional level [11,12] and national level [13]. The estimates of methane emissions from AOG wells have been included in national greenhouse gas inventories of the USA [14] and Canada [15], but still remain largely overlooked in global emissions reports. Although the IPCC sixth assessment report (AR6) [16] mentioned the importance of methane emissions from AOG wells, it failed to give an estimate of the number of AOG wells worldwide and the associated methane emissions. Considering oil and gas reserves [17], drilling history and environmental policies [11], most previous studies have focused on specific regions, which makes it difficult to use them as a basis for generalized conclusions about the mitigation of methane from AOG wells globally. As a result, it is currently difficult to identify effective mitigation actions for high-emitting countries, companies and wells worldwide.

A publicly available, harmonized and comprehensive dataset of methane emissions from AOG wells would help in the evaluation of methane emissions and inform the design of decarbonization interventions in AOG wells across scales, such as at the well level, state level, country level and global level. Here, we first develop a national methane inventory for AOG wells globally, namely the CEADs–Global Abandoned Oil and Gas wells Methane Emissions Inventory (CEADs–AOGI), covering 4.5 million oil and gas wells in 127 countries (with 420 000 AOG wells fully characterized by information about the specific location, type of well and end date). Details of the methods and data used to construct the inventory are shown in the ‘Materials and methods’ section. This inventory sheds light on methane emissions from closed oil and gas activities carried out by specific contributors (countries, companies) and can inform decisions about how to reduce methane emissions in view of global climate targets. Furthermore, we quantify the ‘committed methane emissions’ of existing AOG wells and explore the mitigation potential of plugging the existing AOG wells, based on the year in which the drilling ends (end-spud year), the type of well, the type of terrain and the country in which the well is located. We show the importance of developing net-zero technologies to stop methane emissions from AOG wells.

## RESULTS

The total number of AOG wells worldwide is estimated at 4 499 000, with 3 557 000 wells located in the USA (Fig. S1), according to data that we have collected, harmonized and compiled from various sources, including existing datasets of wells at various scales, research articles and national reports.

### Global distribution of methane emissions from AOG wells

**Figure 1.**
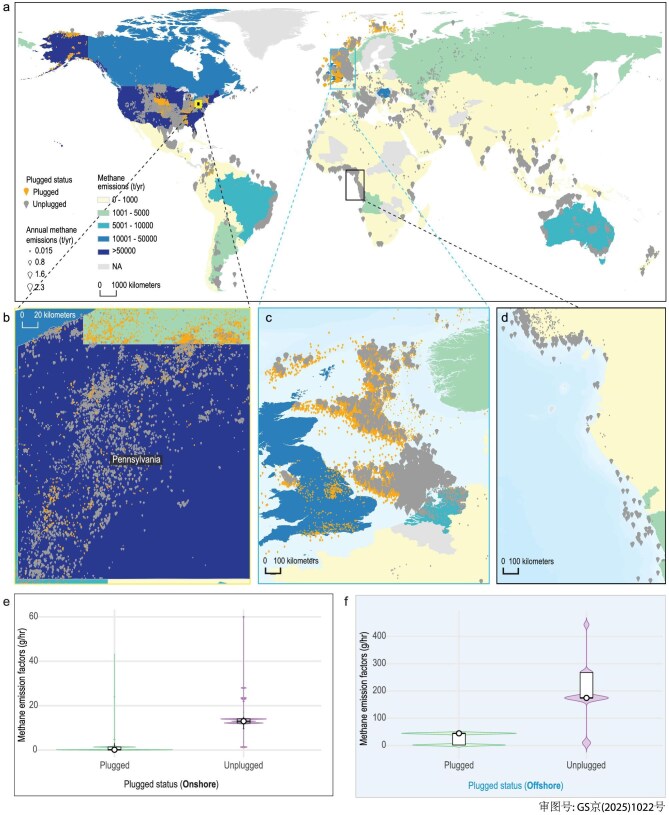
Maps of methane emissions from AOG wells. (a) Global map of methane emissions from AOG wells, with shading indicating the regional distributions of total estimated methane emissions from 4 499 000 wells in 2022. (b–d) Zoomed-in views over (b) Pennsylvania, USA, (c) North Sea Shelf and (d) coastal area of Western Africa, illustrating the distribution of AOG wells. The size of the points represents methane emissions in 2022, categorized as ≤0.01, ≤0.8, ≤1.6 and ≤2.3 t. (e and f) Charts showing the methane-emission factors of plugged and unplugged wells for onshore and offshore AOG wells, respectively. Box plot whiskers indicate variability, with means shown as white circles.

Our emission inventory estimates that methane emissions from 4 499 000 AOG wells worldwide totaled ∼0.4 million tons (Mt) in 2022—equivalent to 10.5 Mt of CO₂ over a 100-year timescale [16] (for details of the coverage of well-level data by region, see Table S1). Figure 1 presents methane emissions from all AOG wells by country in 2022, along with the geographical location and current plugging status of 419 047 AOG wells worldwide.

Methane emissions from AOG wells in the USA were ∼0.2 Mt in 2022, or 70% of the global total. Other major contributors include Canada, Romania, the UK and Brazil, with the top 10 emitting countries collectively responsible for 98% of global emissions (Fig. 1a). This distribution of AOG wells reflects the legacy of historical oil and gas production [17]. The analysis of the 419 047 wells highlights the geographical correlation between intensive historical exploitation and current emission hotspots, emphasizing the long-term environmental impact of past energy production.

Pennsylvania, as an example, with a century-and-a-half-long crude-oil and gas production history [9], accounts for 24% of the methane emissions from US AOG wells in 2022, attributed to its 325 000 wells, many of which remain unplugged (Fig. 1b). Similarly, Texas, with 890 000 AOG wells and a comparable historical production legacy, contributed 22% of the nation's emissions (see details of the AOG numbers in Fig. S1). Although we have collected detailed data about millions of AOG wells, information on numerous orphaned wells is still lacking. Satellite imagery cannot capture sufficiently precise details of these wells, as wells can be covered by overgrown vegetation [18]. These orphaned AOG wells are primarily unplugged and have significantly higher methane-emission potentials: 21 times higher onshore (Fig. 1e) and 8 times higher offshore (Fig. 1f) compared with plugged wells. This calls for enhanced survey efforts to cover orphaned AOG wells, with the aim of improving the accuracy of AOG-well methane inventories and facilitating efficient plugging interventions. Countries such as Romania [19] and Russia face similar challenges, in which orphaned wells with no identifiable operators place a significant management burden on governments.

Offshore super-emitting wells (AOG wells emitting >1.6 t of methane in 2022), with a large fraction concentrated in the North Sea Shelf (Fig. 1c), require targeted investigation due to their disproportionately high methane-emission factors—11 times greater than onshore wells (Fig. 1e and f). In shallow waters (<200 m), methane bubbles from offshore AOG wells are the primary pathway for emissions, especially in the littoral zone, where they are most likely to reach the atmosphere [20]. In deeper waters, only a small amount of emitting methane may reach the atmosphere through turbulent transport [20,21]; however, dissolved methane in the water column can resurface over time due to ocean warming or circulation patterns [22]. Additionally, methane vented directly into the ocean can pollute the water, further disrupting marine ecosystems [23].

### Historical cumulative methane emissions from existing AOG wells

**Figure 2.**
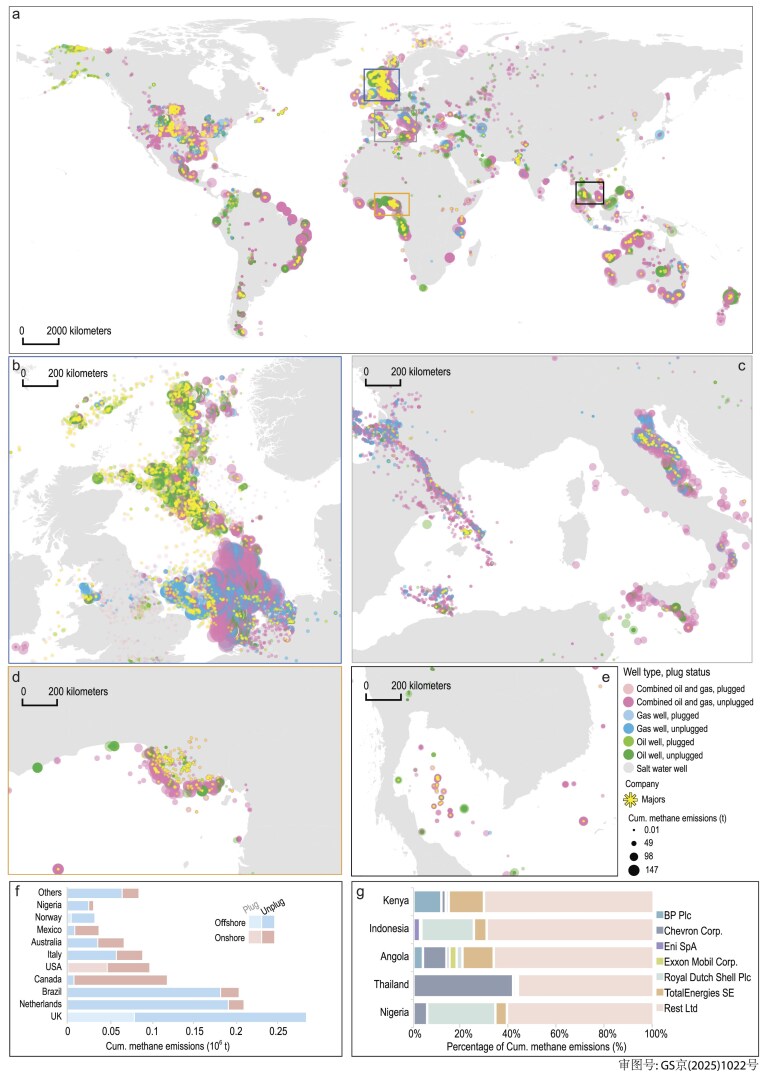
Historical cumulative methane emissions from AOG wells. Map (a) shows the location, well type, plugged status and methane emissions of 419 047 AOG wells worldwide. AOG wells are classified into seven types by resources-type of well, plugged status and cumulative historical methane emissions calculated since wells were abandoned and until the end of 2022 (<0.01, ≤49, ≤98 and >147 t). The color of the dots shows the well type and plugged status; the size of the dots indicates the cumulative methane-emissions amount; sunburst symbols denote wells operated by major oil and gas companies. Insets highlight regional clusters of emissions in (b) the North Sea, (c) the Mediterranean region, (d) the Niger Delta and (e) Southeast Asia. (f) Bar chart showing the distribution of historical cumulative methane emissions from AOG wells across 10 representative countries by well terrain and plugged status. (g) Bar chart showing the contributions of six selected major companies to cumulative methane emissions in typical oil- and gas-producing countries within ASEAN and sub-Saharan Africa.

Given the long history of AOG wells, it is critical to estimate historical cumulative methane emissions from AOG wells. We used a sample of 419 047 AOG wells around the world, with detailed geological and plugging management information, to estimate historical cumulative methane emissions from all existing AOG wells (Fig. 2a). The average age of these AOG wells is 32 years, calculated from the end-spud year to the end of 2022. Our results show that cumulative methane emissions from 4.5 million AOG wells globally are ≥11.2 Mt, of which 99% came from only 10 countries.

Methane emissions from AOG wells, while smaller than those from active wells and other sectors, have unique long-term climate significance. Unlike active wells, which are subject to stricter controls and emit methane only during their operational period, abandoned wells often lack effective oversight and are challenging to monitor with current satellite detection technologies. As a result, these wells can emit methane, a potent greenhouse gas, persistently over decades or even centuries. Historical methane emissions from old AOG wells contribute to the cumulative greenhouse gas effect over time. Although methane does not persist in the atmosphere for as long as CO_2_ [24], it is chemically active [24] and can still contribute to local environmental hazards (e.g. water contamination [25]) and health issues [26] over time. Therefore, accurate accounting of such emissions in global emissions inventories is necessary to ensure a fair distribution of contributions to climate-change mitigation. Addressing these legacy emissions is essential to minimize their long-term impact on global warming [27].

The cumulative methane emissions from offshore AOG wells are partly resulting from massive subsurface methane emissions caused by casing failures, cement degradation and pressure imbalances under high pressure and temperature beneath the seabed [8]. A large fraction of these super-emitting wells is concentrated in the North Sea Shelf (Fig. 2a and b). Unplugged offshore AOG wells in the UK and the Netherlands, according to the latest information gained up to 2022, each account for about 17% of the total (Fig. 2f), though, in the UK, there are more than twice as many offshore AOG wells (3786 wells in the sample) as in the Netherlands (1486 wells in the sample). Clearly, offshore AOG wells represent a persistent source of methane emissions (for the age of AOG wells by region, see Table S2) contributing to marine methane concentration and global warming [22]. Additionally, historically plugged combined oil + gas gas wells in the USA, such as those concentrated on the east coast, which account for 4% of the total US cumulative historical emissions from AOG wells, are still at risk of methane leakage and should be subject to regular monitoring.

Major international oil and gas companies have played a significant role in historical methane emissions from AOG wells, particularly in regions such as sub-Saharan Africa and Southeast Asia (ASEAN, Fig. 2g). Six key companies, out of ∼32 000 companies recorded in the CEADs–AOGI database, are responsible for ∼10% of the cumulative emissions (∼1.3 Mt from the 419 047 wells sampled). For instance, Royal Dutch Shell (Fig. 2g) is a major contributor to methane emissions from AOG wells in Nigeria (the largest oil and gas producer in Africa [28]) and Indonesia (the third-largest oil and gas producer in Asia, Fig. 2g), accounting for 28% and 22% of the cumulative national emissions, respectively. Similarly, Total Energy Group (Fig. 2g) is linked to 13% of Angola's cumulative methane emissions (the second-largest oil-producing country in sub-Saharan Africa [29]). AOG wells once owned by BP Plc contribute 12% of Kenya's cumulative methane emissions from AOG wells (Fig. 2g) whereas 42% of Thailand's cumulative emissions (Fig. 2g) from AOG wells come from wells once owned by Chevron Corporation. Details of the distribution of AOG wells operated by six major companies are shown in Fig. S2. The information about operators reflects the most recent operators of the AOG wells.

### Emission reduction potential of identified AOG wells

As AOG wells vary widely according to age, terrain and well type across regions, region-specific strategies and timelines for plugging efforts [27] are needed. We analyse possible methane-emissions mitigation pathways at the country level, from 2023 to 2050, by optimizing when to plug each AOG well—this analysis is based on four key parameters: country, well terrain, end-spud year and well type (see ‘Materials and methods’ for details about the analysis of the proposed sets of scenarios for methane mitigation).

We project the annual methane emissions from all AOG wells over 2023–50 under three different scenarios, namely a default plugging scenario (the unplugged AOG wells will be completely plugged at their planned plugged year), a 3-years-early plugging scenario (the unplugged AOG wells will be completely plugged 3 years earlier than the scheduled plugged year) and a 3-years-late plugging scenario (the unplugged AOG wells will be completely plugged 3 years later than the scheduled plugged year).

**Figure 3.**
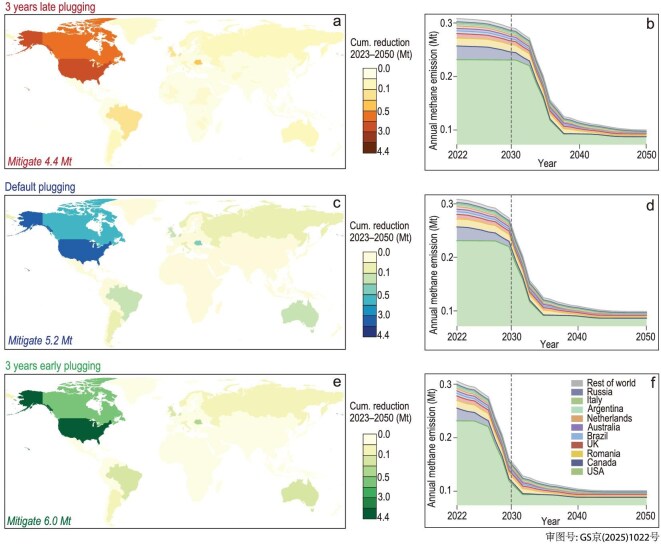
Potential reduction of cumulative methane emissions and annual emissions from all AOG wells over 2023–50. Map of potential reduction of cumulative methane emissions from 2023 to 2050 under the (a) 3-years-late plugging scenario, (c) default plugging scenario and (e) 3-years-early plugging scenario. Trends of annual methane-emissions reduction from AOG wells by key countries under the (b) 3-years-late plugging scenario, (d) default plugging scenario and (f) 3-years-early plugging scenario.

Figure 3 summarizes the potential mitigation of methane emissions from AOG wells over 2023–50. Plugging AOG wells could potentially reduce global cumulative emissions from AOG wells by 53% (default plugging scenario) over 2023–50.

Country-specific management of AOG wells is essential for achieving methane reductions (Fig. 3a, c and e). Of the total projected reductions under the default plugging scenario (5.2 Mt), 70% would come from the USA and 15% from Canada, illustrating the importance of gaining information about all those unplugged AOG wells in North America for which we do not have details (∼2.5 million wells in total) but that if plugged would largely contribute to reduce methane emissions globally. In Europe, Romania contributes 5% (0.3 Mt) of the total reduction potential, largely from its 60 000 onshore unplugged wells. Similarly, the UK, with 92% of its total AOG wells located offshore (3786 unplugged wells), accounts for 3% of the global reduction potential. The high methane-emission factors of offshore wells make their plugging particularly impactful, highlighting the need for prioritization of these high-risk sources.

The average period going from the end of drilling (end-spud year) to the completion of plugging (completely plugged) is ∼22 years based on our global AOG wells dataset.

This delayed timeline introduces substantial methane emissions, known as carbon ‘lock-in’ (Fig. 3b, d and f), which accumulate until intervention occurs. Plugging AOG wells as early as possible can promote large-scale emissions reduction from global AOG wells. From 2023 to 2050, without any plugging efforts of the current unplugged AOG wells, cumulative methane emissions from global AOG wells could reach 9.9 Mt. Such emissions could be compressed to 3.9 Mt with early plugging (3-years-early plugging scenario; see details in ‘Materials and method’), saving 1.6 Mt of emissions space (compared with the 3-years-late plugging scenario) in terms of the carbon budget of the Paris Agreement.

Despite aggressive plugging efforts, residual emissions persist. By 2050, an estimated 0.07 Mt of annual emissions would remain locked in, pointing to the need for developing deep methane-elimination technologies to achieve net-zero emissions.

## DISCUSSION AND CONCLUSIONS

AOG wells are not only a potential significant source of methane emissions, but also represent opportunities for environmental benefits [10]. Although previous studies have estimated the methane emissions from AOG wells in a specific year at the country/region level [11–13], a comprehensive analysis of the actual number, historical methane emissions and spatial distribution of AOG wells worldwide is missing and leaves a sizable gap in the assessment of the national-specific historical methane emissions and the possibility of emissions reduction. Our study addresses these deficiencies by presenting a detailed global inventory of AOG wells and their associated methane emissions. For example, our estimate of annual methane emissions from US AOG wells in 2022, at ∼0.2 Mt CH_4_/year, is more than four times higher than the central value (0.06 Mt CH_4_/year) reported by Alvarez *et al.* [30] and aligns closely with the estimation of 0.3 million Mt of the 2022 inventory of the US EPA [31]. By analysing methane-emissions patterns associated with well type and plugging status, our study provides actionable insights to inform national and global efforts to eliminate emissions from closed oil and gas infrastructure.

In 2022, global methane emissions from AOG wells totaled 0.4 Mt, representing only ∼1% of the methane emissions from active oil and gas production operations [1]. However, this seemingly small percentage highlights a pressing issue: the number of AOG wells continues to grow and, without adequate attention and effective management strategies, their contribution to methane emissions will increase. Despite their significance, most official global emissions reports neglect methane emissions from AOG wells and fail to evaluate the potential for reducing these emissions. This omission hinders the achievement of net-zero climate goals. To address this challenge, methane-elimination technologies and tailored country-specific strategies are essential [3]. Key interventions include the timely plugging of wells, site remediation [32], the development of standardized well inventory frameworks [27] and consistent monitoring of both plugged and unplugged wells. Organizations like the IPCC should consider including methane emissions from AOG wells in their assessment reports and generally focus on investigating the number of global AOG wells to develop a more accurate and standardized assessment of the associated methane emissions.

At the country level, our findings underscore the need for region-specific mitigation strategies based on where the AOG wells are located, the average age, the end-spud year, the type of well and the well terrain. In 2022, the two largest emitters of methane from AOG wells across the world were the USA and Canada. Our analysis has identified a large number of orphaned AOG wells in the USA (2.0 million AOG wells) and Canada (0.4 million AOG wells), which were probably abandoned after the oil-drilling rush between 1859 and the mid-1870s [33]. This number largely exceeds the total number of AOG wells in the rest of the world. As a result, the estimation of methane emissions is largely uncertain and will hinder attempts to adopt methane-reduction strategies. Therefore, quantifying the actual number and investigating the location of historically orphaned AOG wells are critical to reducing methane emissions in North America.

The case of Romania—a major oil-producing country in Europe, with a long history of oil and gas production and many AOG wells—demonstrates the challenges of incomplete well data. Of the estimated 60 000 AOG wells, only 57 have detailed records, with nearly half identified as unplugged offshore wells. Addressing these data gaps is critical for improving methane-reduction efforts and ensuring safety in these regions.

The UK and the Netherlands may be home to old-lasting, super-emitting AOG wells (averaging 28 years, and 43 years since ending spud, respectively, Table S2) concentrated in the North Sea Shelf and must prioritize strategies to manage them in order to achieve methane-emissions reduction, such as (i) improving the transparency of monitoring and reporting offshore AOG wells; (ii) using high-integrity plugs to prevent leakage from wells with higher temperature and pressure underwater; and (iii) providing targeted financial incentives for the proper plugging of offshore AOG wells.

In developing countries in Africa and Asia, many once-foreign-owned oil and gas wells have been abandoned. Collaborative efforts between historical operators and local governments are critical to managing and plugging these wells effectively. Companies such as Royal Dutch Shell, Total Energy and Chevron Corporation, with their greater technical and financial resources [3], have an opportunity to work alongside national governments to mitigate methane emissions and reduce environmental risks.

This study also highlights the role that the management of AOG wells plays in meeting global net-zero goals. For instance, timely plugging of wells could save 1.6 Mt of methane emissions in terms of the carbon budget aligned with the Paris Agreement. However, managing the plugging of AOG wells is not enough. AOG wells that are already plugged can still leak and require deeper cleaning and frequent monitoring. The ongoing energy transition, with an anticipated increase in well decommissioning [34,35], underscores the need for accurate data on the number of oil and gas wells in operation and proposals for drilling and exploration, alongside ways to reduce leakage from AOG wells to achieve future climate goals. The combination of bottom-up investigations, satellite monitoring and Artificial Intelligence technologies offers a promising pathway for quickly identifying orphaned AOG wells, enabling real-time methane-emission monitoring and supporting targeted mitigation actions. To support effective regulatory action, future efforts must prioritize systematic, transparent updates to national AOG well registries. Our findings underscore the importance of investing in the validation of well-level data, particularly in countries with incomplete metadata. Assumptions based solely on public records may result of enlagred uncertainty.

The CEADs–AOGI presented here is subject to uncertainties and limitations. A detailed description of uncertainties is included in the Supplementary information. In general, the average uncertainties of methane emissions are estimated to be 6%–23% for AOG wells in the USA, UK, Netherlands and Norway, with detailed national records, and 13%–23% for AOG wells in the rest of the world. Well-level uncertainties vary among regions, with larger uncertainties for AOG wells without collected government-recorded datasets due to incomplete information. CEADs–AOGI now contains a limited number of AOG wells with detailed information recorded, such as location, end-spud year, plugged status and operator, due to the lack of records of the historical AOG wells, especially the orphaned ones. More state-/country-level data should be collected and incorporated in the future. CEADs–AOGI will be updated and improved as more and better data become available. See Supplementary Section 1.1 for further details of the uncertainty analysis.

## MATERIALS AND METHODS

The CEADs–AOGI developed in this study encompasses 4 499 276 oil and gas wells in 127 countries; 9% of the total wells (419 047 wells) are analysed, with details at the level of the individual well (name, geo-location, status, terrain, start and end date of production), 89% of the total wells (3 994 457 wells) are analysed with information at the state level and, for the remaining 2% (85 772 wells), we only know the country in which the wells are located in.

### Well-based methane-emissions estimation

There are distinctive heterogeneities among AOG wells in terms of terrain, resource type, plugging status, ending spud year, geographical location and so on. We estimate annual methane emissions from AOG wells at the country level according to a well-based accounting methodology as detailed in previous studies [9], using the following equation:


\begin{eqnarray*}
{E}_{c,t} = \sum\limits_{i = 1}^N {E{F}_{i,j,t}},
\end{eqnarray*}

where *c, i, t* and *j* represent the region, wells, year and district (state), respectively. *E* represents the country-level emissions (t), *N* represents the numbers of abandoned wells in each country (t) and *EF* represents the emission factors (t methane emissions per abandoned well). The emission factors differ depending on whether wells are plugged or unplugged, oil + gas mix or gas only, onshore or offshore, and their locations.

#### Number of AOG wells

We compiled AOG-well information from governmental [36–41], regional [42] and industry sources [43,44], complemented with emissions reports [45], relevant news [46,47] and research articles [5,8,9,13]. Where the direct plugging status was unavailable, it was inferred by using national plugging/unplugging ratios. Wells are classified by terrain (onshore/offshore), resource type (oil, gas, oil + gas) and plugging status (plugged, unplugged or partially plugged). Geo-coordinates were standardized to WGS-84. For the unknown information about the terrain of AOG wells, we used the geographical coordinates of the wells to derive information about the terrain (see details of data collection and compilation in Supplementary Section 1.1 and Table S3).

#### Methane-emissions factor

Emission factors vary by region, terrain, resource type and plugging status. Methane-emission factors were compiled from empirical measurements of abandoned wells in previous studies [5,6,8,9,13]. In countries lacking specific data, global average emission factors were applied based on well characteristics. We call for future research on well-/regional-specific emission factors based on experimental measurements with the enhanced satellite observation technologies. Detailed factor inventories are provided in Table S4.

### Proposed sets of scenarios to analyse methane-mitigation paths

To evaluate methane-mitigation strategies, we developed a two-tier scenario framework. Tier 1 defines 24 sub-scenarios based on four parameters: country type (*C*), terrain (*S*), end-spud year (*Y*) and well type (*T*). Tier 2 introduces plugging schedules (*P*), defining whether unplugged wells are sealed on time, 3 years early or 3 years late.

We analysed 6073 US wells with known plugging dates and found an average lag of 22 years from end spud to plugging. This delay was used to model expected plugging timelines globally.

A total of 72 scenarios were constructed to reflect geographic, technical and temporal diversity in plugging and mitigation strategies. The methane-reduction potential was evaluated by using differences in emissions factors between unplugged and plugged wells (for full scenario parameterization, sub-scenario combinations and mitigation-rate calculations, see details in Supplementary Section 1.1).

## Supplementary Material

nwaf184_Supplemental_File

## Data Availability

The numerical results plotted in Figs 1–3 are provided with this paper. We have listed all the data sources and their detailed information in Supplementary Table S3.
