# Correction to: A global inventory of methane emissions from abandoned oil and gas wells and possible mitigation pathways

**DOI:** 10.1093/nsr/nwag152

**Published:** 2026-04-28

**Authors:** Tianyang Lei, Xiujing Chen, Shijun Ma, Dabo Guan, Liang Jing

**Affiliations:** The Bartlett School of Sustainable Construction, University College London, London WC1E 6BT, UK; Department of Earth System Sciences, Tsinghua University, Beijing 100080, China; The Bartlett School of Sustainable Construction, University College London, London WC1E 6BT, UK; The Bartlett School of Sustainable Construction, University College London, London WC1E 6BT, UK; Department of Earth System Sciences, Tsinghua University, Beijing 100080, China; Institute for Carbon Neutrality, Tsinghua University, Beijing 100084, China; Energy Traceability Technology, Technology Strategy and Planning Department, Aramco, Dhahran 31311, Saudi Arabia

In the article by Lei *et al.*, ‘A global inventory of methane emissions from abandoned oil and gas wells and possible mitigation pathways,’ *National Science Review* 2025; **12**: nwaf184. DOI: 10.1093/nsr/nwaf184, an error was identified in Reference 46 (Maintext) and Reference 11 (Supplementary Information), Fig. 2c. We also provide clarification regarding the treatment of Dutch wells, following updated plug-status records released by NLOG.

Error 1: The reference for the estimated number of abandoned wells in Russia (~26,000) was mistakenly attributed to a general news article (Reference 46 in maintext and Reference 11 in Supplementary Information).

Correction 1:

Ирина Скиба, ATinform. *В России могут взорваться около 26 000 заброшенных нефтяных скважин – вице-премьер РФ*. https://atinform.com/news/v_rossii_mogut_vzorvatsja_okolo_26_000_zabroshennykh_neftjanykh_skvazhin_vice_premer_rf/2021-09-09-13374 (Published 9 September 2021).

Error 2: In Fig. 2c, a small number of onshore abandoned oil and gas wells in Italy were incorrectly mapped, appearing in the Pyrenees region. This error resulted from a coordinate reference system (CRS) misalignment during coordinate transformation.

Correction 2: The well data of abandoned oil and gas wells in Italy (shown in Fig. 2c) were sourced from the official UNMIG dataset (data accessed August 22, 2022). The dataset provides coordinates but does not consistently specify CRS metadata for all records. In the original mapping workflow, records lacking CRS information were interpreted as WGS84 by default, which led to an approximately 12.45° longitudinal offset for onshore wells. Diagnostic checks indicate that offshore records are consistent with WGS84, whereas the affected onshore records are consistent with a local Italian CRS (likely Monte Mario/Rome 1940). No source data were altered and no wells were manually repositioned. The issue has been corrected in an updated version of Fig. 2 using proper coordinate transformation. Stricter CRS validation protocols will be adopted in future updates of the CEADs–AOGI database.

Correction 3:

We note that NLOG has updated and clarified plug-status records since our data download, and these updates will be incorporated in the next CEADs–AOGI release. Dutch wells comprise ~0.11% of wells in our inventory and contribute ~1.8% of the global methane estimate. Emission factors were assigned using our globally consistent status-based framework: offshore wells reported as closed-in/abandoned/suspended were assigned 443 g CH_4_ h^−1^(an empirically observed value reported by Schout *et al.*, 2019 for the Netherlands, Ref. 8 in the maintext), while offshore wells explicitly reported as plugged were assigned 0 g CH_4_ h^−1^; onshore wells used the global status–terrain–resource averages due to the lack of Netherlands-specific onshore measurements. Sensitivity tests indicate that alternative plausible treatments of the Dutch subset would change the global total by <1% (≈0.8–0.9%), well within the uncertainty range reported, and do not affect the paper’s conclusions.

We apologize for any inconvenience caused. This correction does not affect the conclusions of the article.

**Figure 2.**
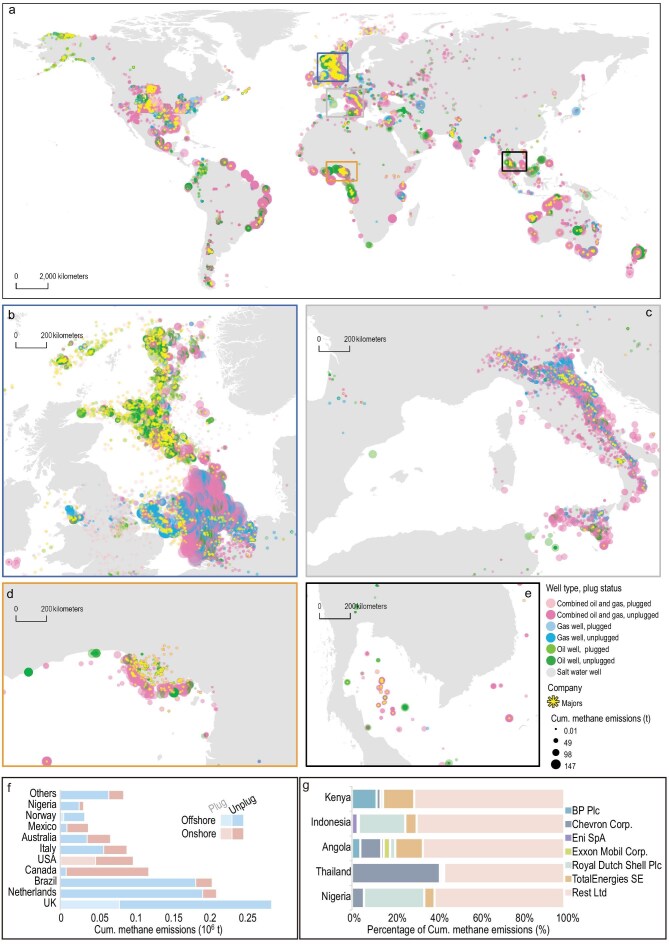
Historical cumulative methane emissions from AOG wells. Map (a) shows the location, well type, plugged status and methane emissions of 419 047 AOG wells worldwide. AOG wells are classified into seven types by resources-type of well, plugged status and cumulative historical methane emissions calculated since wells were abandoned and until the end of 2022 (<0.01, ≤49, ≤98 and >147 t). The color of the dots shows the well type and plugged status; the size of the dots indicates the cumulative methane-emissions amount; sunburst symbols denote wells operated by major oil and gas companies. Insets highlight regional clusters of emissions in (b) the North Sea, (c) the Mediterranean region, (d) the Niger Delta and (e) Southeast Asia. (f) Bar chart showing the distribution of historical cumulative methane emissions from AOG wells across 10 representative countries by well terrain and plugged status. (g) Bar chart showing the contributions of six selected major companies to cumulative methane emissions in typical oil- and gas-producing countries within ASEAN and sub-Saharan Africa.

