# Supplementary material for: A global inventory of methane emissions from abandoned oil and gas wells and possible mitigation pathways
Source: Natl Sci Rev. 2025 May 19;12(7):nwaf184. doi: 10.1093/nsr/nwaf184 (PMC12202153; doi:10.1093/nsr/nwaf184)
Supplement: nwaf184_Supplemental_File [file nwaf184_supplemental_file.docx]

# Supplement information

# Section 1.1 Data collection and compilation

## Number of abandoned oil and gas wells

In this study we define AOG wells those inactive wells with no production, following the definition adopted in previous studies^1^ and national related dataset^2-5^ that included terms such as inactive, suspended, abandoned, orphaned, plugged, plugged back and sidetracked, junked, etc. The AOG wells worldwide are classified by terrain (onshore/offshore), resource type (oil, gas, oil + gas mix), and plugging status (unplugged, plugged, plugged with control) according to previous studies and published datasets. We use three main types of sources (datasets by official institutions, research articles, and relevant news items) to determine the number of AOG wells at the well/state/country level.

We start from the published open-source datasets from the state/national/regional agencies in control of abandoned wells in the United States^4,5^, Canada^6^, The Netherlands^3^, the United Kingdom^2^, and Norway^7^. All dataset sources are listed in *Supplementary Table S3*. Given the high concentration of AOG wells in North America, we have crossed check the state-/plugged status-/ terrain-/resource-type- specific number of AOG wells with previous studies^1,8,9^ covering the US and Canada. We have identified about 3.6 million and 0.8 million AOG wells in the United States and Canada by 2022 respectively; for 44% of them there are recorded details, whereas the remaining 56% are orphaned AOG wells for which state agencies only estimated the number without any detailed well-level information.

Next, we have used the GlobalData oil and gas wells database^10^ to compile the map of abandoned wells located in other countries. As the GlobalData wells database does not provide details about the plugging status of the abandoned wells, for the main oil and gas producing countries, we have searched and used relevant news or reported quotes from the government and have determined the plugging status of a specific number of AOG wells in Russia^11^, Kazakhstan^12^, and Romania^13^. We have also used relevant reports and news to determine the management of abandoned wells in Nigeria^14^, Angola^15^, and Colombia^16^. For unknown plugging status, we use the dataset-wide percentage of unplugged and plugged wells based on the total number of unplugged and plugged wells we have gathered from state/provincial/territorial datasets for that country. For example, in Alaska, US, 96% of wells are plugged, thus, we assumed 96% of the 2533 wells that do not report the plugging status are plugged, while the remaining 4% are unplugged.

## Methane emissions factor

Methane emissions from AOG wells vary with location, terrain, type of resource, plugging status and so on^9^. Historic data of methane emissions have been estimated in several regions, but we have found no comprehensive record for the AOG wells worldwide. We have collected and compiled existing methane emissions factors generated from measurements of abandoned wells in previous studies (e.g., Kang et al. 2014^8^, 2016^9^; Williams et al. 2021^1^, Netherlands^17^ and UK^18^ studies). According to the classification of emissions factors, we have categorized the wells by region, terrain, resource type, and plugging status in the ***CEADs-AOGI*** and have selected the corresponding emissions factors to estimate methane emissions. Details of terrain-/resource-type-/district-/technology- specific methane emission factor inventories for AOG wells worldwide are shown in *Supplementary Table S4*. For AOG wells in countries without published official emissions factors, we have used the global average value generated by the currently known emissions factors classified according to terrain, resource type, and plugging status as the default emissions factor.

We noticed that the records from governments are updated irregularly. For example, information gained from the Nederlandse Olie- en Gasportaal Hoofdnavigatie (NLOG) website in 2022 had limited information about well plugged status. Information we assessed in 2025 included more detailed records. This implies that regular and in-time updates of databases could significantly reduce the uncertainty in methane emission estimates and improve the completeness and reliability of abandoned well inventories.

# Section 1.2 Scenario framework for analyzing methane mitigation pathways

We have collected detailed information about plugged date for 6,073 AOG wells located in the United States. The average period going from the "date of end-spud" to the "date in which plugging is complete" is about 22 years. For the unplugged AOG wells with the details of the dates in which they were closed over the last 22 years, we have assumed they will be plugged 22 years after closure according to their own end-spud date.

For all other unplugged AOG wells, we analyze future methane emissions under two scenario sets that are organized according to a tiered structure. Basically, **Tier 1** scenario sets focus on the country of the AOG wells, the well-terrain, end spud year, and well-type, whereas **Tier 2** scenario sets focus on the choice of actual plugging schedule. We treat each scenario set as the different combination of the individual parameters in the model, such that we will have 2 scenario sets with the combination of five parameters (*C, S, Y, T, P*). The meaning of the five parameters is as follows:

1) ***C*** refers to the countries of the AOG wells; 2) ***S*** refers to the terrain of the AOG wells; 3) ***Y*** refers to the year of end-spud; 4) ***T*** refers to the type of AOG wells. 5) ***P,*** a parameter in Tier 2, refers to the actual plugging year of the unplugged AOG wells.

We vary the value of each parameter by considering different sub-scenarios within each scenario set. This analysis aims to investigate the effects of the different portfolios of methane mitigation options and their impacts in terms of methane reductions from the AOG wells globally.

**Tier 1 includes 2 scenario sets:**

**Scenario set 1: the planned year of plugging.** Scenario set 1 defines the planned year of the plugging of each AOG wells according to 4 parameters: the countries where AOG wells are located, terrain of the AOG wells, the end spudding year, and the type of AOG wells.

**Parameter 1: Countries where the AOG wells are located.** Parameter set 1 defines the country in which the AOG wells are located. The acronym *C* means 'Country of AOG well'. We assume that plugging will happen first in the most developed countries**.**

- ***C*1**: developed countries.
- ***C2***: high-income developing countries.
- ***C3***: other developing countries.

**Parameter 2: Terrain of the AOG wells.** Parameter set 2 defines the terrain of AOG wells. The acronym *S* means 'Terrain of AOG well'.

- ***S*1**: Onshore wells.
- ***S2:*** Offshore wells.

**Parameter 3: Year of the end-spud.** Parameter set 3 defines the end-spud year of AOG wells. The acronym *Y* means 'the Year of the end-spud' and varies across the AOG wells worldwide.

**Parameter 4: Type of AOG wells.** Parameter set 4 defines the type of AOG wells. The acronym *T* means 'Type of AOG well'.

- ***T*1**: oil wells.
- ***T2***: combined oil and gas wells.
- ***T3***: gas wells.
- ***T4***: other type of wells.

The combination of each variation of the above 4 parameter sets gives a distribution strategy. We will have 3 x 2 x 1 x 4 =24 sub-scenarios in scenario set 1.

**Tier 2 includes 1 scenario set:**

**Scenario set 2: Plugging schedule of AOG wells.** This scenario determines the plugging year of each AOG well according to one parameter. The acronym *P* means 'plugging schedule'.

Parameter set 2 defines the actual plugging year of each AOG well. The planned plugging date is the year by which the AOG well reaches the average service life of its terrain (**Parameter *S***) and country (**Parameter *C***) since the end spudding (**Parameter *Y***). Based on the planned plugging date, AOG wells will be retrofitted under 2 sub-scenarios:

- ***P*1** (Default): the AOG wells will be plugged at the planned plugging date.
- ***P*2** (Three years early plugging case): the AOG wells will be plugged three years ahead of the planned plugging date.
- ***P*3** (Three years late plugging case): the AOG wells will be plugged three years later than the planned plugging date.

The combination of each variation of the above 2 parameters gives a distribution strategy. We will have 3 sub-scenarios in scenario set 2.

The combination of each variation of the above 5 parameters gives a distribution strategy. In this analysis, we will have 3 x (3 x 2 x 1 x 4) = 72 scenarios.

We have calculated the region (continental shelf, country, state)-/terrain-/well-type specific methane mitigation rate of plugging AOG wells, according to the ratio of methane emission factors between plugged wells and unplugged wells.

# Supplement Figures

## Figure S1 Number of AOG wells worldwide

The total number of AOG wells worldwide are estimated at 4,499,000 of which (3,557,000 wells) are in the United States (**Figure S1**) according to our collected and compiled well/state/territorial datasets, research articles, national reports.


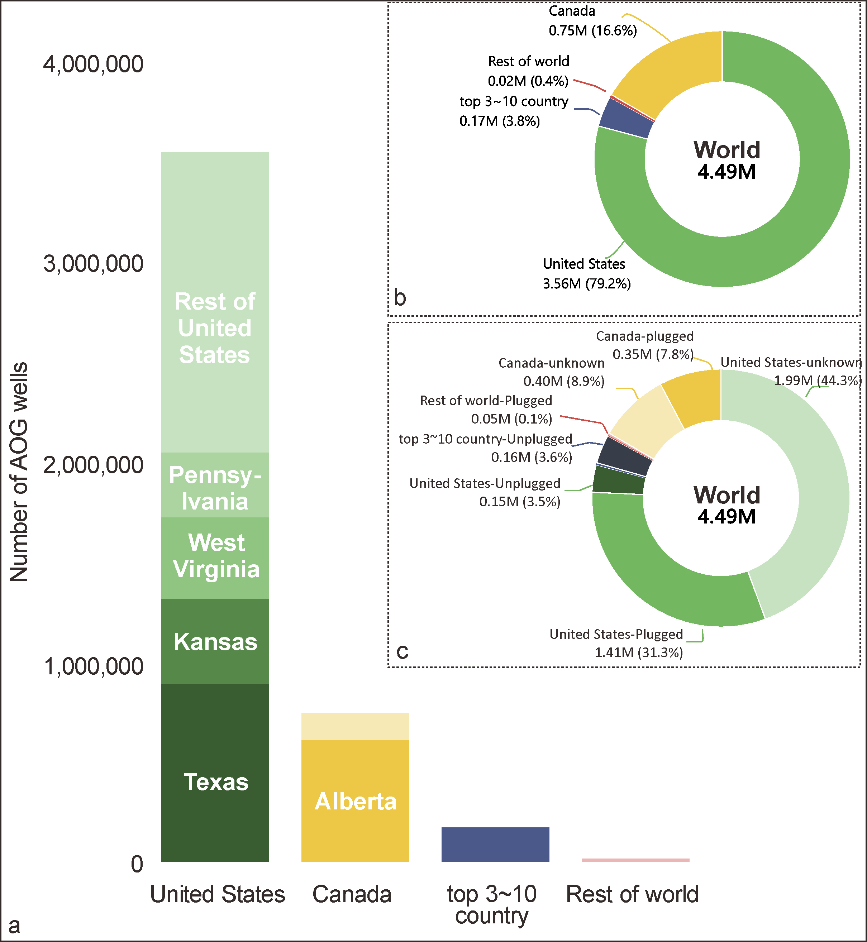


**Figure S1** **Number and distribution of AOG wells worldwide. a,** Number of the AOG wells by state/country/region; **b,** proportion of the AOG wells by main regions; **c,** proportion of the AOG wells by plugging-status at the region-level. top 3~10 country is classified by the number of national AOG wells: Romania, Argentina, Russia, Australia, United Kingdom, Brazil, Norway, and Mexico. Labels in the column represent the names of the state.

Globally, **abandoned oil and gas wells are highly concentrated in a few countries** due to the oil and gas reserves and production history^19^. We find that up to 99.6% (4476.1 thousands) of the total number of AOG wells worldwide in 2022 are located in a mere ten countries (**Figure S1**).

Specifically, **United States owns more than 79.2% of global AOG wells**, mainly located in Texas, Kansas, West Virginia, and Pennsylvania, with century-and-a-half-long crude oil and gas production history^9^, accounting for 25.1%, 12.0%, 11.6%, 9.1% of the national total, respectively (**Figure S1**a). Canada is the second-largest controller of AOG wells worldwide, owning 746.9 thousand AOG wells, of which 81.7% locate in Alberta. **The high spatial concentration of AOG wells indicates that the management of abandoned oil and gas wells in these states is critical to the methane emissions from inactive oil and gas operations, not only for their own country but also globally**. This has forced these state governments to investigate and obtain clear statistics on AOG wells: accurate number, location, and management policies, to reduce the uncertainty of AOG wells (lasting methane leakage, risks from sink), which may also benefit the applications for the storage potential of these inactive bore holes in the future. Moreover, the importance of Romania, one of the largest and oldest producers of oil and gas in Europe^20^ cannot be ignored, having about 60 thousand AOG wells, most of which were drilled without modern environmental standards, making them not only a significant source of methane leakage but may also be very dangerous. Thus, it is an urgent need for Romania government companies to tackle action on the management of AOG wells.

**Figure S1**c shows the plugging status of the recorded global AOG wells by region. Obviously, **the proportion of plugged wells of the currently known AOG wells in the United States is higher than in the other region** (90% of total domestic known 1.6 million AOG wells), reflecting the strict management of discovered AOG Wells in the United States. A high proportion of plugged wells to the domestic total also shows in Canada, with 46.7% of its AOG wells have been plugged. Conversely, the general plugging rate in the other countries is lower than 30%, with 6.5% for the top 3~10 countries, and 27.1% for the rest of the world, calling for an urgent investigation and clearance of those abandoned oil and gas wells in the future.

Despite the higher plugging rate, **a vast amount of AOG wells remain unknown in the United States and Canada, which may be left from the oil drilling rush between 1859 and the mid-1870s**^21^. This number is even more than 12 times the total AOG wells in the rest of the world, urging these two countries to target these historically left orphaned oil and gas wells to reduce methane leakage and the associated high uncertainties.

## Figure S2 Maps of historical cumulative methane emissions from AOG wells owned by the largest six oil and gas companies by well type and plugged status


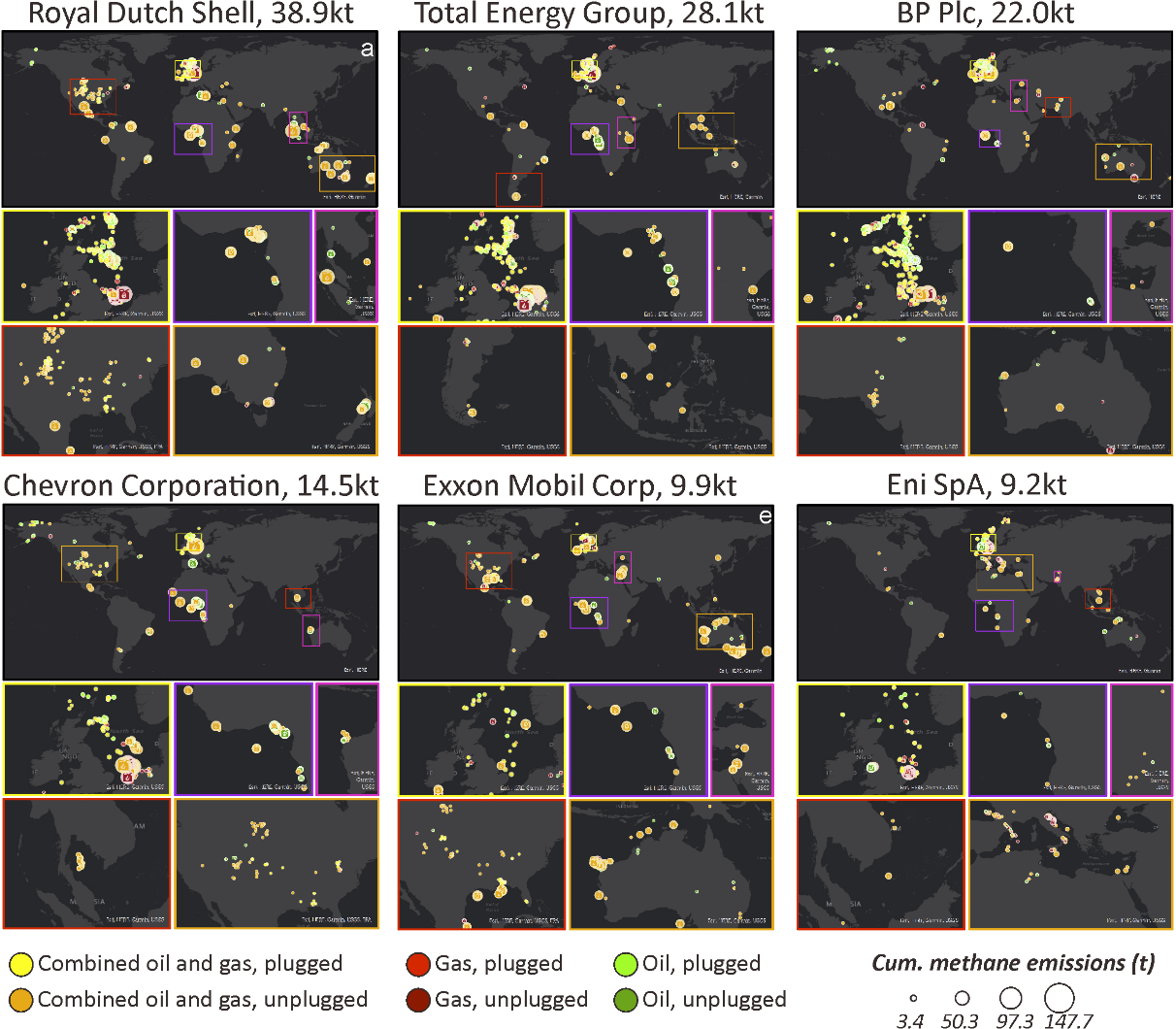


**Figure S2 Maps of historical cumulative methane emissions from AOG wells owned by the largest six oil and gas companies by well type and plugged status**. (a)-(f), Location of AOG wells owned by the largest six oil and gas companies, Royal Dutch Shell (a), Total Energy Group (b), BP Plc (c), Chevron Corporation (d), Exxon Mobil Corp (e), Eni SpA (f). AOG wells are classified into 7 types by type of well, plugged status and cumulative historical methane emissions calculated since wells were abandoned and until end of 2022 (<3.4t, ≤50.3t, ≤97.3t, >147.7t). The color of the dots shows the well type and plugged status; the size of the dots indicates the cumulative methane emissions amount. Text in each column tags the name of the operator and the cumulative emissions from the samples AOG wells.

# Supplement Tables

## Table S1 Data coverage of the abandoned oil and gas (AOG) wells by region

| Region | Number of AOG wells with well-level details | Number of AOG wells at the country-level |
| --- | --- | --- |
| United States | 286,352 | 3,556,595 |
| Canada | 22,653 | 746,867 |
| Argentina | 32,625 | 32,625 |
| Australia | 21,362 | 21,362 |
| Brazil | 9,439 | 9,439 |
| Netherlands | 5,128 | 5,128 |
| Italy | 7,246 | 7,246 |
| Romania | 57 | 60,000 |
| Russia | 263 | 26,000 |
| United Kingdom | 12,562 | 12,562 |
| Rest of world | 21,360 | 21,452 |
| Total | 419,047 | 4,499,276 |

## Table S2 Age of the AOG wells by region

| Region | Average age in 2022 (duration years since end spudding to 2022) |
| --- | --- |
| United States | 25.0 |
| Canada | 50.3 |
| Argentina | 27.4 |
| Australia | 14.5 |
| Brazil | 36.3 |
| Netherlands | 43.0 |
| Romania | 24.7 |
| Russia | 40.6 |
| United Kingdom | 27.7 |
| Rest of world | 34.5 |

## Table S3 Data sources of number of AOG wells and the methane emission factor

| **country** | **region** | **year** | **Data sources of AOG Number** | **Data sources of plugged details** | **Data available level** | **Notes** |
| --- | --- | --- | --- | --- | --- | --- |
| Russia | Whole country | 2021 | Gaspard Sebag, et al. ^11^ |  | country-level | EF using default; Assume all unknown sector wells as MIX_ABANDON |
| Kazakhstan | Whole | 2021 | «Казинформ» ^12^ |  | country-level | EF using average value of previous literatures in United States and Canada, Williams,2021; Assume all unknown sector wells as MIX_ABANDON |
| Romania | Whole | 2014 | Popescu ^13^ |  | country-level | EF using default; Assume all unknown sector wells as MIX_ABANDON |
| United Kingdom | Whole | 2016 | Oil and Gas Authority ^2^ |  | well-level | EF using default; Assume all unknown sector wells as MIX_ABANDON |
| Norway | Whole | 2022 | Norwegian petroleum directorate ^7^ |  | well-level |  |
| Netherlands | Whole | 2021 | NLOG ^3^ |  | well-level |  |
| United States | Pennsylvania | 2022 | DEP Office ^4^ |  | well-level |  |
| United States | Oklahoma | 2022 | Oklahoma Corporation Commission ^22^ |  | well-level |  |
| United States | Ohio | 2021 | Ohio Department of Natural Resources ^23^ |  | well-level |  |
| United States | New York | 2021 | New York State Department of Environmental Conservation^24^ |  | well-level |  |
| United States | Alabama | 2022 | State of Alabama Oil and Gas Board^25^ |  | well-level |  |
| United States | Alaska | 2022 | Alaska Oil & Gas Conservation Commission^26^ |  | well-level |  |
| United States | Arizona | 2020 | Kang, et al. ^27^ |  | state-level |  |
| United States | Aekansas | 2020 | Williams, et al. ^1^ |  | state-level |  |
| United States | California | 2020 | Williams, et al. ^1^ |  | state-level |  |
| United States | Colorado | 2020 | Williams, et al. ^1^ |  | state-level |  |
| United States | Florida | 2020 | Williams, et al. ^1^ |  | state-level |  |
| United States | Idaho | 2020 | Williams, et al. ^1^ |  | state-level |  |
| United States | Illinois | 2020 | Williams, et al. ^1^ |  | state-level |  |
| United States | Indiana | 2020 | Williams, et al. ^1^ |  | state-level |  |
| United States | Iowa | 2020 | Kang, et al. ^27^ |  | state-level |  |
| United States | Kansas | 2020 | Williams, et al. ^1^ |  | state-level |  |
| United States | Kentucky | 2020 | Williams, et al. ^1^ |  | state-level |  |
| United States | Louisiana | 2020 | Williams, et al. ^1^ |  | state-level |  |
| United States | Maryland | 2020 | Williams, et al. ^1^ |  | state-level |  |
| United States | Michigan | 2020 | Williams, et al. ^1^ |  | state-level |  |
| United States | Mississippi | 2020 | Williams, et al. ^1^ |  | state-level |  |
| United States | Missouri | 2020 | Williams, et al. ^1^ |  | state-level |  |
| United States | Montana | 2020 | Williams, et al. ^1^ |  | state-level |  |
| United States | Nebraska | 2020 | Williams, et al. ^1^ |  | state-level |  |
| United States | Nevada | 2020 | Williams, et al. ^1^ |  | state-level |  |
| United States | New Mexico | 2020 | Williams, et al. ^1^ |  | state-level |  |
| United States | North Dakota | 2020 | Williams, et al. ^1^ |  | state-level |  |
| United States | Oregon | 2020 | Williams, et al. ^1^ |  | state-level |  |
| United States | South Dakota | 2020 | Kang, et al. ^27^ |  | state-level |  |
| United States | Tennessee | 2020 | Williams, et al. ^1^ |  | state-level |  |
| United States | Texas | 2020 | Williams, et al. ^1^ |  | state-level |  |
| United States | Utah | 2020 | Williams, et al. ^1^ |  | state-level |  |
| United States | Virginia | 2020 | Williams, et al. ^1^ |  | state-level |  |
| United States | Washington | 2020 | Williams, et al. ^1^ |  | state-level |  |
| United States | West Virginia | 2020 | Williams, et al. ^1^ |  | state-level |  |
| United States | Wyoming | 2020 | Williams, et al. ^1^ |  | state-level |  |
| Canada | Alberta | 2020 | Kang, et al. ^27^ |  | state-level |  |
| Canada | British Columbia | 2020 | Williams, et al. ^1^ |  | state-level |  |
| Canada | Manitoba | 2020 | Williams, et al. ^1^ |  | state-level |  |
| Canada | New Brunswick | 2020 | Williams, et al. ^1^ |  | state-level |  |
| Canada | Nova Scotia | 2020 | Williams, et al. ^1^ |  | state-level |  |
| Canada | Ontario | 2020 | Kang, et al. ^27^ |  | state-level |  |
| Canada | Quebec | 2020 | Williams, et al. ^1^ |  | state-level |  |
| Canada | Saskatchewan | 2020 | Williams, et al. ^1^ |  | state-level |  |
| Canada | Yukon | 2020 | Williams, et al. ^1^ |  | state-level |  |
| Canada | Northwest Territories | 2020 | Williams, et al. ^1^ |  | state-level |  |
| Canada | Prince Edward Island | 2020 | Williams, et al. ^1^ |  | state-level |  |
| Italy | Whole country | 2022 | UNMIG^28^ |  | well-level | EF selected as the average value of North Sea |
| Algeria | Whole country | 2022 | GlobalData ^10^ |  | well-level | No regulations on the management of abandoned oil and gas wells were found, thus, we assume all the abandoned oil and gas wells in Algeria were abandoned without plug. |
| Angola | Whole country | 2022 | GlobalData ^10^ | Lusa ^15^ | well-level | All found abandoned oil and gas wells are decommissioned prior to 2015, as the new strict regulations on plugged inactive wells come into power in 2018, so we assumed all the recorded abandoned oil and gas wells in Angola were unplugged. 2) the Angola government are investing in finding the abandoned oil and gas fields to avoid further dangerous^29^ |
| Colombia | Whole country | 2022 | GlobalData ^10^ | DUQUE ^16^ | well-level | Wells closed after 2009 (include 2009) supposed to be plugged according to the related regulations in 2009, while wells closed prior to 2009 supposed to be unplugged, excepted the wells operated by Ecopetrol SA. |
| Nigeria | Whole country | 2022 | GlobalData ^10^ | Business & Maritime West Africa ^14^ | well-level |  |
| Rest of world | All the other countries | 2022 | GlobalData ^10^ |  | well-level | Global average value of known emission factors |

## Table S4 Methane emission factor inventories for global AOG wells

| **Country** | **Constituent Entity** | **Unit** | **O&G-unplugged** | **gas-unplugged** | **all unplugged** | **O&G-plugged** | **gas-plugged** | **all plugged** | **Source** |
| --- | --- | --- | --- | --- | --- | --- | --- | --- | --- |
| Canada | Alberta | methane g/hr | 14 | 15 | 12 | 0.051 | 4.8 | 0.0018 | Williams et al.,2021^1^ |
| Canada | British Columbia | methane g/hr | 12 | 22 | 0.15 | 0.046 | 4.8 | 1.5 | Williams et al.,2021^1^ |
| Canada | Manitoba | methane g/hr | 12 | 28 | 9.6 | 0.12 | 18 | 2.5 | Williams et al.,2021^1^ |
| Canada | Newfoundland and Labrador | methane g/hr | 12 | 28 | 9.6 | 0.12 | 18 | 2.5 | Williams et al.,2021^1^ |
| Canada | Northwest Territories | methane g/hr | 14 | 15 | 12 | 0.051 | 4.8 | 0.0018 | Williams et al.,2021^1^ |
| Canada | Nova Scotia | methane g/hr | 12 | 28 | 9.6 | 0.12 | 18 | 2.5 | Williams et al.,2021^1^ |
| Canada | Saskatchewan | methane g/hr | 14 | 15 | 12 | 0.051 | 4.8 | 0.0018 | Williams et al.,2021^1^ |
| Canada | Unknown | methane g/hr | 12 | 22 | 10 | 0.046 | 4.8 | 1.5 | Williams et al.,2021^1^ |
| Canada | Rest of Canada | methane g/hr | 12 | 22 | 10 | 0.046 | 4.8 | 1.5 | Williams et al.,2021^1^ |
| United States | Pennsylvania | methane g/hr | 12 | 60 | 22 | 0.33 | 24 | 15 | Kang et al.,2019^9^ |
| United States | Pennsylvania | methane g/hr | 0.19 | 60 | 22 | 0.33 | 24 | 15 | Kang et al.,2019^9^ |
| United States | Alabama | methane g/hr | 14 | 28 | 9.6 | 0.13 | 18 | 2.8 | Williams et al.,2021^1^ |
| United States | Alaska | methane g/hr | 13 | 17 | 15 | 0.051 | 4.8 | 0.0018 | Williams et al.,2021^1^ |
| United States | Arkansas | methane g/hr | 14 | 28 | 9.6 | 0.13 | 18 | 2.8 | Williams et al.,2021^1^ |
| United States | California | methane g/hr | 13 | 17 | 15 | 0.051 | 4.8 | 0.0018 | Williams et al.,2021^1^ |
| United States | Central Planning Area | methane g/hr | 13 | 23 | 11 | 0.51 | 4.8 | 1.6 | Williams et al.,2021^1^ |
| United States | Colorado | methane g/hr | 13 | 23 | 11 | 0.51 | 4.8 | 1.6 | Williams et al.,2021^1^ |
| United States | Eastern Planning Area | methane g/hr | 14 | 28 | 9.6 | 0.13 | 18 | 2.8 | Williams et al.,2021^1^ |
| United States | Florida | methane g/hr | 14 | 28 | 9.6 | 0.13 | 18 | 2.8 | Williams et al.,2021^1^ |
| United States | Illinois | methane g/hr | 14 | 28 | 9.6 | 0.13 | 18 | 2.8 | Williams et al.,2021^1^ |
| United States | Indiana | methane g/hr | 14 | 28 | 9.6 | 0.13 | 18 | 2.8 | Williams et al.,2021^1^ |
| United States | Kansas | methane g/hr | 13 | 17 | 15 | 0.051 | 4.8 | 0.0018 | Williams et al.,2021^1^ |
| United States | Kentucky | methane g/hr | 14 | 28 | 9.6 | 0.13 | 18 | 2.8 | Williams et al.,2021^1^ |
| United States | Louisiana | methane g/hr | 14 | 28 | 9.6 | 0.13 | 18 | 2.8 | Williams et al.,2021^1^ |
| United States | Michigan | methane g/hr | 14 | 28 | 9.6 | 0.13 | 18 | 2.8 | Williams et al.,2021^1^ |
| United States | Mississippi | methane g/hr | 14 | 28 | 9.6 | 0.13 | 18 | 2.8 | Williams et al.,2021^1^ |
| United States | Montana | methane g/hr | 13 | 17 | 15 | 0.051 | 4.8 | 0.0018 | Williams et al.,2021^1^ |
| United States | Nevada | methane g/hr | 13 | 17 | 15 | 0.051 | 4.8 | 0.0018 | Williams et al.,2021^1^ |
| United States | New Mexico | methane g/hr | 13 | 17 | 15 | 0.051 | 4.8 | 0.0018 | Williams et al.,2021^1^ |
| United States | New York | methane g/hr | 14 | 28 | 9.6 | 0.13 | 18 | 2.8 | Williams et al.,2021^1^ |
| United States | North Dakota | methane g/hr | 13 | 17 | 15 | 0.051 | 4.8 | 0.0018 | Williams et al.,2021^1^ |
| United States | Ohio | methane g/hr | 14 | 28 | 9.6 | 0.13 | 18 | 2.8 | Williams et al.,2021^1^ |
| United States | Oklahoma | methane g/hr | 14 | 22 | 17 | 0.51 | 4.8 | 1.6 | Williams et al.,2021^1^ |
| United States | Pacific Outer Continental Shelf Offshore | methane g/hr | 13 | 23 | 11 | 0.51 | 4.8 | 1.6 | Williams et al.,2021^1^ |
| United States | Pennsylvania | methane g/hr | 12 | 48 | 21 | 0.17 | 18 | 9.6 | Williams et al.,2021^1^ |
| United States | Tennessee | methane g/hr | 14 | 28 | 9.6 | 0.13 | 18 | 2.8 | Williams et al.,2021^1^ |
| United States | Texas | methane g/hr | 13 | 22 | 16 | 0.51 | 4.8 | 1.6 | Williams et al.,2021^1^ |
| United States | Rest of United States | methane g/hr | 13 | 23 | 11 | 0.51 | 4.8 | 1.6 | Williams et al.,2021^1^ |
| United States | Utah | methane g/hr | 13 | 23 | 11 | 0.51 | 0.041 | 0.024 | Williams et al.,2021^1^ |
| United States | Virginia | methane g/hr | 14 | 28 | 9.6 | 0.13 | 18 | 2.8 | Williams et al.,2021^1^ |
| United States | West Virginia | methane g/hr | 13 | 23 | 3.2 | 0.51 | 4.8 | 0.1 | Williams et al.,2021^1^ |
| United States | Western Planning Area | methane g/hr | 13 | 17 | 15 | 0.051 | 4.8 | 0.0018 | Williams et al.,2021^1^ |
| United States | Wyoming | methane g/hr | 13 | 17 | 15 | 0.051 | 4.8 | 0.0018 | Williams et al.,2021^1^ |
| United States | Rest of west states in United States | methane g/hr | 1.7 | 1.7 | 1.7 | 0.002 | 0.002 | 0.002 | Small et al.,2016^30^ |
| United States | Rest of eest states in United States | methane g/hr | 28.0 | 28.0 | 28.0 | 0 | 0 | 0 | Small et al.,2016^30^ |
| United States | Coalbed AOG wells in the United States | methane g/hr | 0.0011 | 5.2 | 1.2 | 0.000012 | 47 | 43 | Small et al.,2016^30^ |
| United Kingdom | Whole country | methane g/hr | 3.6 | 3.6 | 3.6 | 1.7 | 1.7 | 1.7 | Boothroyd et al.,2016^18^ |
| United Kingdom | Offshore_average | methane g/hr | 267.6 | 267.6 | 267.6 | 44.6 | 44.6 | 44.6 | Böttner et al.,2020^31^ |
| Netherlands | Whole country | methane g/hr | 443 | 443 | 443 | 0 | 0 | 0 | Schout et al.,2019^17^ |
| Norway | Offshore_average | methane g/hr | 174.5 | 174.5 | 174.5 | 1.8 | 28.5 | 1.8 | Vielstädte et al.,2015^32^ |
| North Sea | Offshore_average | methane g/hr | 174.5 | 174.5 | 174.5 | 30.8 | 30.8 | 30.8 | Böttner et al.,2020^31^ |
| Rest of World | Offshore_average | methane g/hr | 174.5 | 174.5 | 174.5 | 30.8 | 30.8 | 30.8 | Böttner et al.,2020^31^ |
| Rest of World | Onshore_average | methane g/hr | 12.3 | 23.5 | 11.4 | 0.2 | 11.0 | 3.1 | Average value of the onshore wells |

# References in Supplementary Materials

1 Williams, J. P., Regehr, A. & Kang, M. Methane Emissions from Abandoned Oil and Gas Wells in Canada and the United States. *Environ Sci Technol* **55**, 563-570, doi:10.1021/acs.est.0c04265 (2021).

2 Authority, O. a. G. (ed Oil and Gas Authority) (2016).

3 NLOG. (ed NLOG) (2022).

4 Office, D. (ed Pennsylvania Department of Environmental Protection) (2022).

5 Agency, U. S. E. P. Inventory of U.S. Greenhouse Gas Emissions and Sinks (2022).

6 Canada, E. a. C. C. National inventory report 1990-2020: Greenhouse gas sources and sinks in Canada. (Environment and Climate Change Canada, 2022).

7 directorate, N. p. (ed Norwegian petroleum directorate) (2022).

8 Kang, M. *et al.* Direct measurements of methane emissions from abandoned oil and gas wells in Pennsylvania. *Proc Natl Acad Sci U S A* **111**, 18173-18177, doi:10.1073/pnas.1408315111 (2014).

9 Kang, M. *et al.* Identification and characterization of high methane-emitting abandoned oil and gas wells. *Proc Natl Acad Sci U S A* **113**, 13636-13641, doi:10.1073/pnas.1605913113 (2016).

10 GlobalData. (ed GlobalData) (2022).

11 Gaspard Sebag, Simon Lee & Bloomberg. *Russian oligarchs flocked to the Bay of Billionaires and Cap d’Antibes. Now their French Riviera mansions are in the sanctions spotlight*, <<https://fortune.com/2022/03/28/russia-oligarchs-bay-of-billionaires-cap-dantibes-french-riviera-mansions-sanctions-spotlight/>> (2022).

12 «Казинформ», М. *Когда ликвидируют аварийные нефтяные скважины в Казахстане* <<https://nangs.org/news/upstream/kogda-likvidiruyut-avarijnye-neftyanye-skvazhiny-v-kazakhstane>> (2021).

13 Popescu, M. *OMV Petrom nu-şi asumă pericolul sondelor abandonate în Prahova*, <<https://www.observatorulph.ro/economic/38117-omv-petrom-nu-si-asuma-pericolul-sondelor-abandonate-in-prahova>> (2014).

14 Africa, B. M. W. *32 Oil Wells Abandoned In Niger Delta* <<https://businessandmaritimewestafrica.com/32-oil-wells-abandoned-in-niger-delta/#:~:text=Shell%2C%20is%20said%20to%20have%20abandoned%2032%20of,Akwa%20Ibom%20State%20for%20increased%20daily%20oil%20production>.> (2012).

15 Lusa. *Angola aperta regras no abandono de poços de petróleo*, <<https://www.dn.pt/lusa/angola-aperta-regras-no-abandono-de-pocos-de-petroleo-9284993.html>> (2018).

16 DUQUE, M. S. *Ecopetrol incumplió normas en abandono de pozo con derrame*, <<https://www.eltiempo.com/justicia/investigacion/ecopetrol-incumplio-normas-en-abandono-de-pozo-con-derrame-en-barrancabermeja-198376>> (2018).

17 Schout, G., Griffioen, J., Hassanizadeh, S. M., Cardon de Lichtbuer, G. & Hartog, N. Occurrence and fate of methane leakage from cut and buried abandoned gas wells in the Netherlands. *Sci Total Environ* **659**, 773-782, doi:10.1016/j.scitotenv.2018.12.339 (2019).

18 Boothroyd, I. M., Almond, S., Qassim, S. M., Worrall, F. & Davies, R. J. Fugitive emissions of methane from abandoned, decommissioned oil and gas wells. *Sci Total Environ* **547**, 461-469, doi:10.1016/j.scitotenv.2015.12.096 (2016).

19 IEA. The Oil and Gas Industry in Energy Transitions. (2020).

20 Menoud, M. *et al.* CH4 isotopic signatures of emissions from oil and gas extraction sites in Romania. *Elementa: Science of the Anthropocene* **10**, doi:10.1525/elementa.2021.00092 (2022).

21 Hinton, D. D. & Olien, R. M. *Oil in Texas: The Gusher Age, 1895–1945*. (University of Texas Press, 2002).

22 Commission, O. C. (ed Oklahoma Corporation Commission) (2022).

23 Resources, O. D. o. N. (ed ODNR - Division of Oil & Gas) (2022).

24 Conservation, N. Y. S. D. o. E. (ed Division of Mineral Resources New York State Department of Environmental Conservation) (2021).

25 Board, S. o. A. O. a. G. (ed State of Alabama Oil and Gas Board) (2022).

26 Department of Commerce, C., and Economic Development Alaska Oil and Gas Conservation Commission. (ed Alaska Oil & Gas Conservation Commission) (2022).

27 Kang, M. *et al.* Orphaned oil and gas well stimulus—Maximizing economic and environmental benefits. *Elementa: Science of the Anthropocene* **9**, doi:10.1525/elementa.2020.20.00161 (2021).

28 (UNMIG), I. e. g. (2022).

29 Costa, T. *ANPG gasta rês milhões em estudo para localizar blocos petrolíferos abandonadaos no país*, <<https://www.verangola.net/va/pt/072022/Energia/31865/ANPG-gasta-tr%C3%AAs-milh%C3%B5es-em-estudo-para-localizar-blocos-petrol%C3%ADferos-abandonados-no-pa%C3%ADs.htm>> (2022).

30 Townsend‐Small, A., Ferrara, T. W., Lyon, D. R., Fries, A. E. & Lamb, B. K. Emissions of coalbed and natural gas methane from abandoned oil and gas wells in the United States. *Geophysical Research Letters* **43**, 2283-2290, doi:10.1002/2015gl067623 (2016).

31 Böttner, C. *et al.* Greenhouse gas emissions from marine decommissioned hydrocarbon wells: leakage detection, monitoring and mitigation strategies. *International Journal of Greenhouse Gas Control* **100**, doi:10.1016/j.ijggc.2020.103119 (2020).

32 Vielstädte, L. *et al.* Quantification of methane emissions at abandoned gas wells in the Central North Sea. *Marine and Petroleum Geology* **68**, 848-860, doi:10.1016/j.marpetgeo.2015.07.030 (2015).
